# Supplementary material for: Two-year-olds’ visual exploration of response options during memory decisions predicts metamemory monitoring one year later
Source: Nat Commun. 2025 Jun 11;16:5284. doi: 10.1038/s41467-025-60273-8 (PMC12159179; doi:10.1038/s41467-025-60273-8)
Supplement: Supplementary file 1 — Supplementary Information [file 41467_2025_60273_MOESM1_ESM.pdf]

Two-year-olds’ Behavioral Indices of Memory Evaluation Predict the Emergence of Metamemory Monitoring One Year Later

Supplementary Table 1: Correlations Between Variables in Path Models

Measure	1	2	3	4	5	6	7	8	9	10	11	12	13
**1. Time 1 Gaze Transitions**	___	.20	-.07	-.04	-.23	.16	.19	-.12	.00	.01	-.10	-.05	.17
**2. Time 1 Response Latencies**	.041	___	-.09	-.05	.01	.13	.11	.07	.01	.15	.25	-.08	-.15
**3. Time 1 Accuracy**	.427	.294	___	.09	.18	-.04	.04	.19	.09	-.04	-.07	-.19	.26
**4. Time 1 “ I don’ t know” Rating**	.660	.591	.235	___	.09	-.14	.06	.18	.31	.03	.10	-.24	.09
**5. Time 1 Age**	.008	.913	.023	.235	___	-.03	.00	-.02	-.02	-.13	.50	-.11	.01
**6. Time 2 Gaze Transitions**	.115	.201	.646	.119	.742	___	.23	.14	-.04	.15	.24	-.20	.03
**7. Time 2 Response Latencies**	.063	.282	.648	.544	.964	.018	___	-.12	.03	.01	.00	-.06	-.05
**8. Time 2 Accuracy**	.205	.440	.023	.029	.795	.104	.197	___	.02	.08	.22	-.07	.08
**9. Time 2 Mental State Language**	.978	.915	.298	<.001	.819	.653	.727	.792	___	-.03	.09	-.04	.11
**10. Time 2 Theory of Mind**	.879	.088	.636	.696	.109	.095	.943	.319	.690	___	.06	.08	-.20
**11. Time 2 Age**	.296	.005	.392	.202	<.001	.006	.959	.008	.312	.432	___	-.10	-.02
**12. Time 2 Average Confidence**	.636	.404	.037	.008	.201	.032	.498	.458	.648	.394	.253	___	-.02
**13. Time 2 Metamemory Monitoring**	.097	.148	.005	.334	.937	.795	.600	.377	.248	.027	.831	.849	___

Pearson correlation coefficients (top half of table, above the diagonal) and significance values of these correlation coefficients (bottom half of table, below the diagonal) between all variables across path models. All tests were two-tailed. No adjustments were made for multiple comparisons.

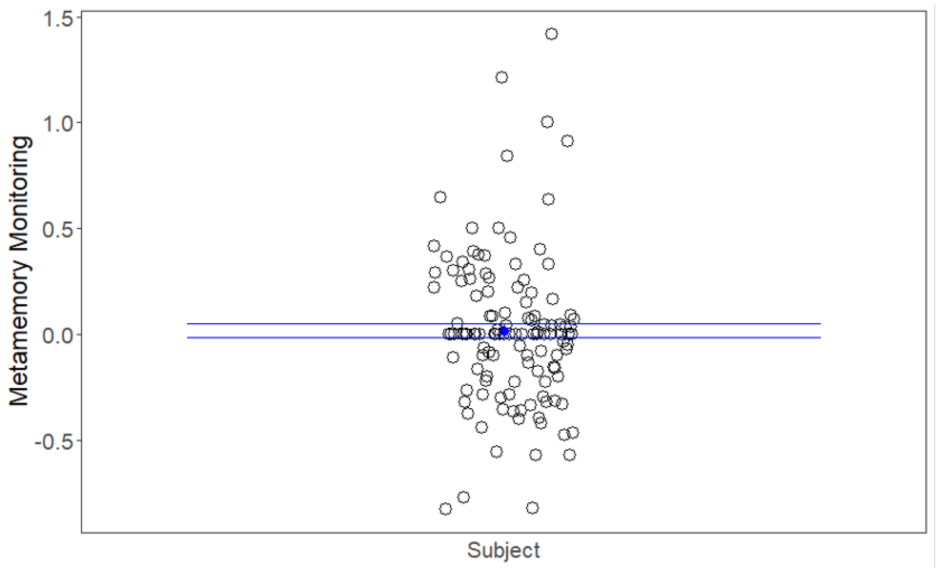

Supplementary Fig. 1. **Distribution of Metamemory Monitoring Scores**. Scatterplot of the Time 2 metamemory monitoring scores (n = 123). Metamemory monitoring was calculated by taking the average confidence score for the participants accurate responses and subtracting their average confidence for inaccurate responses. Positive scores indicate good metamemory monitoring ability, whereas negative scores indicate poor metamemory monitoring abilities. Error bar is the standard error around the mean.

Supplementary Note 1

Exploration of Connections between Response Latencies, Gaze Transitions, Accuracy, and Confidence.

Given the insignificant results of concurrent Time 2 response latencies and gaze transitions to metamemory monitoring, we decided to explore the relations between these variables a little bit more to determine whether there were any connections between them.

First, we explored the connections between Time 2 response latencies, confidence, and accuracy. In our main longitudinal path model from the main manuscript, average response latencies at Time 2 were not associated with confidence at the same time point, nor was the first order correlation significant (r = -.06, p = .498, 95% confidence interval (CI) = -.24 - .12; Supplementary Table 1). Therefore, we decided to do some exploratory analyses for the 51 3-year-olds who showed evidence of metamemory monitoring (confidence for accurate trials *minus*

confidence for inaccurate trials > 0), to determine whether there were any connections between response latencies and confidence when the children actually have the ability to more accurately judge their confidence levels for memory decisions. We first examined whether these children showed the expected relation between response latencies and metamemory monitoring such that children who had better metamemory monitoring discrimination (higher metamemory monitoring scores) would show a bigger differentiation between response latencies for inaccurate trials compared to accurate trials (i.e., longer response latencies for inaccurate compared to accurate trials). Therefore, we calculated a response latency difference score (average response latencies for inaccurate trials minus average response latencies for accurate trials) and correlated this score with their metamemory score. We found that there was a correlation (r = .27, p = .054, CI = -.00 - .51), such that children who had higher metamemory scores also had bigger response latency difference scores. To further explore this, we conducted a multiple regression model, predicting metamemory monitoring from the response latency difference score, while controlling for memory accuracy and age. We found that both the response latency difference score (*B* = .32, p = .012, CI = .07 - .57) and memory accuracy (*B* = .49, p < .001, CI = .23 - .74) independently predicted metamemory monitoring. Thus, although gaze transitions at 3-years of age are generally associated with overall lower confidence (main manuscript finding), among children with stronger metamemory monitoring, the measure of response latency discriminability between accurate and inaccurate trials was also relevant.

Next, we explored the connections between gaze transitions, accuracy, and confidence at Time 2. The main longitudinal model revealed a concurrent association between Time 2 gaze transitions and Time 2 average confidence, such that 3-year-olds who responded less confidently overall were more likely to show more gaze transitions (β = -.19, SE = .03, *p* = .044, CI = -.37 - -

.01; Figure 2). By exploring the individual correlations for gaze transitions for inaccurate and accurate trials separately we found that the association is primarily driven by inaccurate trials ($r = -.20$, $p = .041$, CI = -.38 - -.01 for inaccurate trials; $r = -.17$, $p = .076$, CI = -.34 - .02 for accurate trials). We confirmed this pattern with a multi-level approach to examine data at the trial level. We conducted a Poisson multilevel model predicting gaze transitions from trial level confidence ratings. We found that for trials in which the children selected "not so sure" as their confidence rating, gaze transitions were significantly higher compared to trials in which children selected "really sure" as their confidence rating, $b = .16$, $z = 2.56$, $p = .011$, CI = .04 - .28. This indicates that gaze transitions are more informative about subjective confidence across the entire sample and that they may have still informed decision confidence judgments at Time 2, even though average gaze transitions at Time 2 were not related to children's ability to differentiate metamemory monitoring ability (i.e., the ability to differentiate between accurate and inaccurate responses with their confidence).

Supplementary Note 2

Pre-registered Theory of Mind and Mental State Language Path Models.

In the main manuscript, we added our theory of mind (ToM) and mental state language variables all together into one model with our gaze transition and response latency variables. However, in our pre-registration (https://osf.io/9wz2m) we described two separate models. Therefore, originally, we conducted two separate path models for ToM and mental state language. In order to keep the main manuscript as concise as possible, and because it revealed the same results, we elected to report the combined model, however, for transparency we wished to also report the individual models here in the Supplementary Materials.

Our first model added Time 2 ToM to our main predictors of gaze transitions, response latencies, and accuracy. We also included paths predicting Time 2 ToM from Time 1 gaze transitions and response latencies. This model had overall good fit (robust $X^2(15) = 20.94$, p = .139, robust RMSEA = .06, and robust CFI = .93). Similar to the model in the main manuscript, the path from Time 2 ToM and Time 2 metamemory monitoring was not significant (β = -.17, SE = .09, p = .055, CI = -.34 - -.01). Replicating the results of the first model, we found significant longitudinal paths from Time 1 gaze transitions to Time 2 metamemory monitoring, β = .24, SE= .03, p = .007, CI = .07 - .41, from Time 1 accuracy to Time 2 metamemory monitoring, β = .28, SE= .30, p = .023, CI = .08 - .49, and from Time 1 accuracy to Time 2 accuracy, β = .20, SE= .11, p = .048, CI = .01 - .39. The path from Time 1 response latencies to Time 2 metamemory monitoring was no longer significant, but followed the same trend as the earlier model, β = -.17, SE= .02, p = .052, CI = -.34 - .01.

The second model added in Time 1 "I don't know" usage and Time 2 mental state language to our main predictors of gaze transitions, response latencies, and accuracy. We also included general language ability at Time 1 as an additional covariate to ensure that longitudinal relations did not depend on overall differences in the variability in general language. Finally, we also included paths predicting Time 2 mental state language from Time 1 gaze transitions and response latencies. This model had overall poor fit (robust $X^2(23) = 45.47$, p = .003, robust RMSEA = .09, and robust CFI = .87). Similar to the model in the main manuscript, the paths between Time 1 "I don't know" ratings and Time 2 metamemory monitoring and between Time 2 mental state language and Time 2 metamemory monitoring were not significant (β = .08, SE= .02, p = .335, CI = -.08 - .24; β = .19, SE= .05, p = .058, CI = .00 - .37). There was also a significant path from Time 1 general language to Time 2 mental state language, β = .44, SE= .24,

p < .001, CI = .30 - .58 and a significant concurrent path from Time 1 general language to Time 1 use of the "I don't know" expression, β = .43, SE= .36, p < .001, CI = .31 - .55. Replicating the results of the first model in the main manuscript, we also found significant longitudinal paths from Time 1 gaze transitions to Time 2 metamemory monitoring, β = .25, SE= .03, p = .005, CI = .08 - .42, from Time 1 accuracy to Time 2 metamemory monitoring, β = .29, SE= .30, p = .016, CI = .09 - .49, from Time 1 response latencies to Time 2 metamemory monitoring, β = .21, SE= .02, p = .024, CI = -.40 - -.02, and from Time 1 accuracy to Time 2 accuracy, β = .20, SE= .11, p = .047, CI = .01 - .39.

Therefore, similar to the combined model that was reported in the main manuscript, it does not seem like awareness of ignorance at age 2 years and concurrent ToM are related to the emergence of metamemory monitoring in 3-year-olds.

Supplementary Note 3

Path Model with Conditionalized Theory of Mind Variable Coding.

In light of the fact that there are different ways to code accuracy in theory of mind tasks, we decided to explore an additional coding scheme which scores were conditionalized by accuracy in the control check question[2]. In this coding method, we gave credit for passing each version of the false-belief task only to children who responded correctly to the control check question in that version. We then took the average across these new accuracy scores to get the final theory of mind task accuracy. We then replaced our previous theory of mind scores for this new score in the path model and examined the results. Fit for this model was again poor (robust $X^2(27) = 46.75$, p = .011, robust RMSEA = .08, and robust CFI = .89). Similar to the model in the text, the paths between Time 1 "I don't know" ratings and Time 2 metamemory monitoring and between Time 2 ToM and Time 2 metamemory monitoring were not significant (β = .07,

SE= .02, *p* = .400, CI = -.09 - .23; *β* = -.05, SE= .08, *p* = .542, CI = -.20 - .11). However, we did find a significant path from Time 2 mental state language to Time 2 metamemory monitoring that was not significant in the previous model, *β* = .20, SE= .05, *p* = .048, CI = .01 - .38. All other significant paths investigated in the main manuscript remained significant (Time 1 gaze transitions to metamemory monitoring: *β* = .25, SE= .03, *p* = .004, CI = .08 - .42; Time 1 accuracy to metamemory monitoring: *β* = .29, SE= .30, *p* = .016, CI = .09 - .49; Time 1 Response Latencies to Time 2 metamemory monitoring: *β* = -.21, SE= .02, *p* = .024, CI = -.40 - -.02; Time 1 accuracy to Time 2 accuracy: *β* = .20, SE= .11, *p* = .049, CI = .01 - .39).

Supplementary References

1. Sobel, D. M., & Austerweil, J. L. Coding choices affect the analyses of a false belief measure. *Cognitive Development*, **40**, 9-23 (2016).
