# Supplementary material for: Two-year-olds’ visual exploration of response options during memory decisions predicts metamemory monitoring one year later
Source: Nat Commun. 2025 Jun 11;16:5284. doi: 10.1038/s41467-025-60273-8 (PMC12159179; doi:10.1038/s41467-025-60273-8)
Supplement: Supplementary file 2 — Reporting Summary [file 41467_2025_60273_MOESM2_ESM.pdf]

nature portfolio

Corresponding author(s): Dr. Sarah Leckey
Dr. Simona Ghetti

Last updated by author(s): Apr 8, 2025

Reporting Summary

Nature Portfolio wishes to improve the reproducibility of the work that we publish. This form provides structure for consistency and transparency in reporting. For further information on Nature Portfolio policies, see our Editorial Policies and the Editorial Policy Checklist.

Statistics

For all statistical analyses, confirm that the following items are present in the figure legend, table legend, main text, or Methods section.

n/a	Confirmed	
☐	☒	The exact sample size (n) for each experimental group/condition, given as a discrete number and unit of measurement
☐	☒	A statement on whether measurements were taken from distinct samples or whether the same sample was measured repeatedly
☐	☒	The statistical test(s) used AND whether they are one- or two-sided<br>*Only common tests should be described solely by name; describe more complex techniques in the Methods section.*
☐	☒	A description of all covariates tested
☐	☒	A description of any assumptions or corrections, such as tests of normality and adjustment for multiple comparisons
☐	☒	A full description of the statistical parameters including central tendency (e.g. means) or other basic estimates (e.g. regression coefficient) AND variation (e.g. standard deviation) or associated estimates of uncertainty (e.g. confidence intervals)
☐	☒	For null hypothesis testing, the test statistic (e.g. F, t, r) with confidence intervals, effect sizes, degrees of freedom and P value noted<br>*Give P values as exact values whenever suitable.*
☒	☐	For Bayesian analysis, information on the choice of priors and Markov chain Monte Carlo settings
☒	☐	For hierarchical and complex designs, identification of the appropriate level for tests and full reporting of outcomes
☐	☒	Estimates of effect sizes (e.g. Cohen's d, Pearson's r), indicating how they were calculated

Our web collection on statistics for biologists contains articles on many of the points above.

Software and code

Policy information about availability of computer code

Data collection	The eye tracker task was administered on a Tobii T-120 17-in eye-tracker monitor. Experiments were coded using Tobii Studio, version 3.0.9. For the touchscreen task experiments were coded using Direct RT version 2006.2.0.28.
Data analysis	All statistical analyses were done using R version 4.4.2. R packages used for data analysis included lavaan (version 0.6-19), effsize (version 0.8-1), lsr (version 0.5-2), ggplot2 (version 3.5.1), and glmmTMB (version 1.1.10). All custom code developed for this study is available in the Open Science Framework Repository, https://osf.io/mz3xj/.

For manuscripts utilizing custom algorithms or software that are central to the research but not yet described in published literature, software must be made available to editors and reviewers. We strongly encourage code deposition in a community repository (e.g. GitHub). See the Nature Portfolio guidelines for submitting code & software for further information.

Data

Policy information about availability of data

All manuscripts must include a data availability statement. This statement should provide the following information, where applicable:

- Accession codes, unique identifiers, or web links for publicly available datasets
- A description of any restrictions on data availability
- For clinical datasets or third party data, please ensure that the statement adheres to our policy

The datasets generated and analyzed during the current studies are available in the Open Science Framework repository, https://osf.io/mz3xj/.

Research involving human participants, their data, or biological material

Policy information about studies with human participants or human data. See also policy information about sex, gender (identity/presentation), and sexual orientation and race, ethnicity and racism.

Reporting on sex and gender	Findings were not examined to determine whether they were different between sexes and sex was not considered in study design, other than to attempt to recruit equal numbers of males and females. Sex was determined by parental reporting in our demographics form. Across our entire sample we had 93 females and 83 males. We did not examine sex-based differences in our sample for our analyses because we did not have a big enough sample size for these types of analyses.
Reporting on race, ethnicity, or other socially relevant groupings	We had parents report on race and ethnicity for the child. The categories listed on the demographics form included; white, Asian, African American, Pacific Islander, and other. The other category then asked the parents to fill our the relevant race information. Additionally, we have a separate question which asked whether they were hispanic or non-hispanic. For any children that identified with multiple races, we created the multi-racial category.
Population characteristics	See below
Recruitment	Participants were recruited from a database of names of families who had previously expressed interest in participating in child development studies. These families were originally contacted about interest when the child was an infant by state birth records.
Ethics oversight	University of California, Davis IRB

Note that full information on the approval of the study protocol must also be provided in the manuscript.

Field-specific reporting

Please select the one below that is the best fit for your research. If you are not sure, read the appropriate sections before making your selection.

☐ Life sciences ☒ Behavioural & social sciences ☐ Ecological, evolutionary & environmental sciences

For a reference copy of the document with all sections, see nature.com/documents/nr-reporting-summary-flat.pdf

Behavioural & social sciences study design

All studies must disclose on these points even when the disclosure is negative.

Study description	The study used a quantitative longitudinal design.
Research sample	A sample of 176 toddlers aged 25-34 months (M = 29 months, 93 female) participated in the first assessment (Time 1). Time 2 occurred on average 12.41 months (SD = 3.42) later and included 157 children from Time 1, now preschoolers, aged 35-60 months (M = 41.47 months, 84 female). Parents reported their children's race as White (N = 119), Asian (N= 5), African American (N = 5), multi-racial (N = 37), and unreported (N=10). Parents of 31 children identified them as Hispanic. Families' household incomes were less than $15,000 (N= 5), $15,000-$25,000 (N = 5), $25,000-$40,000 (N = 15), $40,000-$60,000 (N = 29), $60,000-$90,000 (N = 33), more than $90,000 (N = 85) and unreported (N = 4). The children were recruited from a Northern California community, from a database of families contacted from birth records, who had expressed interest in participating in child development studies. This may have introduced some self-selection bias, as families had to initially tell us at first contact whether they were interested in being in the database at all. Therefore, the families that we contacted for this study had already participated in other studies for other labs on campus and were more likely to participate. This may have biased our demographic sample slightly but overall it is fairly representative of the community in which the study was conducted.
Sampling strategy	The sample was a convenience sample. The sample size was based on the original grant proposal for the main analyses. That target sample size was 150 which was deemed be sufficient to have a power of.80 to identify close fit in the proposed structural models using root mean squared error of approximation and alpha = .05 as indication of close fit in the null model.
Data collection	An eye-tracking computer and touch screen computer were utilized for data collection of the variables. Additional control variables utilized small toys and objects. In addition, parents filled out language and demographic surveys with pen and paper. Children sat on the parents lap during the eye-tracking task and for the touchscreen task, only the experimenter and the child were present. Experimenters collecting the data were blinded to study hypotheses. No other blinding was utilized.
Timing	Data collection occurred between October 2014 and July 2019.
Data exclusions	Time 1: Not all participants contributed data in both tasks due to some toddlers being uncooperative in one (33) or both tasks (6), not returning for all sessions (3), computer malfunction (8), they completed an advanced pilot version of the memory task (9), or a combination of these reasons (2). Therefore, 133 toddlers contributed eye movements data and 145 toddlers contributed response latency data. Before calculating the accuracy and gaze switch variables for the eye-tracker version, we first eliminated trials for which children did not provide an answer. This resulted in 57 trials (2.12%) across 18 toddlers being eliminated from analyses. Next, we removed retrieval trials for which the toddlers had not looked at the picture during the encoding phase, indicated by no fixations during the 3-second period of encoding phase. This criterion resulted in the exclusion of 155 trials (5.77%) across 68 toddlers. Finally, we also removed trials for which the eye-tracker did not measure any look time to either AOI, target or distractor during the retrieval

phase. This criterion resulted in the exclusion of 415 trials (15.45%) across 80 toddlers being eliminated from analyses. Once these eliminations were made, accuracy and average gaze transitions for each participant were calculated. Additionally, after average gaze transitions were calculated, the distribution of the variable was examined and several potential outliers were visually seen. Therefore, we removed any average gaze transitions that were +/- 3 standard deviations from the mean. This resulted in 1 average gaze transition score being removed. Before calculating the accuracy and response latency variables from the touchscreen version, similar to the eye-tracker version, we removed trials for which children did not provide an answer. This resulted in 10 trials (.36%) across 6 toddlers being eliminated. Next, we removed any trials with response latencies less than 700 ms in duration. These trials were likely to be responses produced before processing the stimuli or trials in which the toddler was inattentive. We followed previous research in toddlers which has used 700 ms as a touching response latency response cutoff21. This criterion resulted in 20 trials (.72%) across 14 toddlers being eliminated from analyses. Finally, we also removed trials where the z-scored response latencies across each individual participant were +\- 3 standard deviations. This resulted in 71 trials (2.56%) across 71 toddlers being eliminated from analyses. Once these eliminations were made, average response latencies and accuracy for each participant were calculated. After average response latencies were calculated, three scores were removed for being +/- 3 standard deviations away from the mean.
Time 2: Not all participants at Time 2 contributed data in both tasks due to being uncooperative in one (15) or both tasks (3), computer issues (9), not returning for all sessions (3), ran out of time during the session (2), or they completed an advanced pilot version of the touchscreen task (4). Therefore, 134 preschoolers contributed eye movements data and 128 preschoolers contributed response latency and confidence data. Similar to Time 1, trials were removed before analysis for not providing answers, quick response latencies, and no looking time measured to stimuli. For response latencies, 3 trials (.12%) across 3 participants were removed due to preschoolers not providing a confidence rating and no trials were eliminated due to preschoolers not providing an answer. Nine trials (.36%) across 7 participants were removed for responses being below the 700 ms cutoff. Sixty-three trials (2.50%) across 63 preschoolers were eliminated due to our outlier cutoff of 3 SD above or below the individuals mean. Once average response latencies were calculated, four scores were removed due to being +/- 3 standard deviations away from the mean. For gaze transitions, there were 74 trials (2.76%) across 22 preschoolers which were eliminated due to preschoolers not providing an answer. There were 399 trials (14.86%) across 98 participants removed due to preschoolers not looking at the image during encoding. Finally, there were 304 trials (11.32%) across 83 preschoolers eliminated from analysis due to no looking time measured towards the target or the distractor. No average scores were removed for being outliers of +/- 3 standard deviations away from the mean.

Non-participation

Reasons for participants not returning for Time 2 included our inability to contact them (10), moved out of area (4), and no longer interested in participating (5).

Randomization

There was no manipulation, so no randomization was required.

Reporting for specific materials, systems and methods

We require information from authors about some types of materials, experimental systems and methods used in many studies. Here, indicate whether each material, system or method listed is relevant to your study. If you are not sure if a list item applies to your research, read the appropriate section before selecting a response.

Materials & experimental systems

n/a	Involved in the study	
☒	☐	Antibodies
☒	☐	Eukaryotic cell lines
☒	☐	Palaeontology and archaeology
☒	☐	Animals and other organisms
☒	☐	Clinical data
☒	☐	Dual use research of concern
☒	☐	Plants

Methods

n/a	Involved in the study	
☒	☐	ChIP-seq
☒	☐	Flow cytometry
☒	☐	MRI-based neuroimaging

Plants

Seed stocks

NA

Novel plant genotypes

NA

Authentication

NA
